# Assessment of the Risk of Foot and Mouth Disease among Beef Cattle at Slaughter from East African Production Systems

**DOI:** 10.3390/v13122407

**Published:** 2021-12-01

**Authors:** Julie Adamchick, Karl M. Rich, Andres M. Perez

**Affiliations:** 1 Department of Veterinary Population Medicine, College of Veterinary Medicine, University of Minnesota, Minneapolis, MN 55108, USA; aperez@umn.edu; 2 Department of Agricultural Economics, Ferguson College of Agriculture, Oklahoma State University, Stillwater, OK 74078, USA; karl.rich@okstate.edu

**Keywords:** risk assessment, foot and mouth disease, commodity-based trade, Kenya, Uganda

## Abstract

Endemic foot and mouth disease (FMD) in East African cattle systems is one factor that limits access to export markets. The probability of FMD transmission associated with export from such systems have never been quantified and there is a need for data and analyses to guide strategies for livestock exports from regions where FMD remains endemic. The probability of infection among animals at slaughter is an important contributor to the risk of FMD transmission associated with the final beef product. In this study, we built a stochastic model to estimate the probability that beef cattle reach slaughter while infected with FMD virus for four production systems in two East African countries (Kenya and Uganda). Input values were derived from the primary literature and expert opinion. We found that the risk that FMD-infected animals reach slaughter under current conditions is high in both countries (median annual probability ranging from 0.05 among cattle from Kenyan feedlots to 0.62 from Ugandan semi-intensive systems). Cattle originating from feedlot and ranching systems in Kenya had the lowest overall probabilities of the eight systems evaluated. The final probabilities among cattle from all systems were sensitive to the likelihood of acquiring new infections en route to slaughter and especially the probability and extent of commingling with other cattle. These results give insight into factors that could be leveraged by potential interventions to lower the probability of FMD among beef cattle at slaughter. Such interventions should be evaluated considering the cost, logistics, and tradeoffs of each, ultimately guiding resource investment that is grounded in the values and capacity of each country.

## 1. Introduction

Livestock and animal products comprise a large portion of the economy for East African countries, including Kenya and Uganda. The livestock sector of Kenya contributes 12% of national gross domestic product and employs approximately 10 million people; 60% of households in Kenya own livestock [1]. In Uganda, livestock accounts for 4.3% of national gross domestic product (GDP), 58% of households own livestock, and 92% of those are subsistence farmers [2]. The vast majority of livestock revenue is from domestic sales with a small fraction sold to neighboring countries through formal and informal channels. If each country were able to expand into international markets and increase the sales volume and margins received for animal-source goods, these countries could improve livelihoods for participants in the agricultural sector and strengthen the infrastructure that supports animal health, production, and food safety. For these reasons, market access can be viewed as a tool toward economic growth as well as improved public health and food security [3].

Several challenges hinder the profitable and sustainable supply of East African livestock to premium international markets. One barrier is that countries will not import animal-source goods that could carry diseases that threaten their own animal or public health. Foot and mouth disease (FMD) is a highly contagious disease of cattle and other livestock and wildlife species, which has been eradicated from many parts of the world but remains endemic in much of Africa and Asia [4,5]. There is a tremendous economic cost associated with outbreaks of FMD in naïve populations of agricultural animals (cost estimates for past outbreaks range from 0.5–10 billion US dollars) because of impacts on animal health and productivity, costs to control the disease, and knock-on repercussions for the affected country’s participation in international trade [6].

In regions where FMD remains endemic, local conditions make it very difficult to eliminate. Many countries lack robust veterinary infrastructure and institutions [7,8,9]; systemic issues are compounded by the difficulties of animal surveillance and vaccination in remote areas [10]. The challenges described as most significant to the control of FMD in Africa in 2016 [11] are largely unchanged from those identified in 1982 [12]. In contrast to South America, where FMD control efforts have been largely successful and vaccine coverage is roughly 146% (i.e., all cattle are vaccinated more than once a year on average), it is estimated that only 5.5% of African cattle are vaccinated annually [6].

Kenya and Uganda have both tried unsuccessfully to establish disease-free zones in which FMD and other transboundary diseases of livestock would be controlled, monitored, and eventually eliminated for the sake of enabling international exports. The areas designated by Uganda as Disease Control Zones in 2011 [13,14] are still the foci of beef development projects, but have since pivoted to emphasize production efficiency, environmental sustainability, and value addition rather than achieving freedom from disease [15]. In Kenya’s Vision2030 [16], one of the goals for economic development through agriculture was to establish four disease-free zones for export, hoping to expand disease freedom to include a large swath of the country by 2022. To date, some progress has been made toward individual export zones, which would function more as quarantine stations than disease-free regions [17], though construction on the most advanced was called off in June 2020 due to apparent lack of progress by the contractor [18].

Standards established by the OIE (World Organisation for Animal Health) underpin transactions between World Trade Organization Member Countries related to animal health [19] and provide strategies for trade from regions infected with FMD. Options include establishing disease-free compartments, processing goods such that the virus would be destroyed, or demonstrating that the risk of transmission via the product to be traded is reduced to an acceptable level (according to international standards and/or the requirements of the importing country) [20]. In the latter case, known as commodity-based trade, established protocols for commodity-based trade recognized by OIE countries in practice have not yet been established. The risk of transmission associated with the final product is influenced by the geographic presence of FMD in the region but is also impacted by actions pre- and post-harvest to detect, eliminate, and prevent contamination with FMD virus, together with a well-documented and traceable process [21]. Scientific risk assessment of the threat posed by a product is the method recognized by the OIE to justify protective trade measures by importing countries [22]. Risk assessments are an essential tool for demonstrating the fitness of one’s goods for the international marketplace as well as for understanding and improving animal and public health domestically [23]. 

There is little information available to complete a risk assessment for beef produced from East African cattle systems. The risk reduction achieved by post-harvest steps of inspection, processing, and storage of deboned beef according to OIE specifications has been extensively reviewed and assessed [21]. Their analysis, starting from the assumption that 100% of animals arriving to the facility were infected with FMD, was that the risk of FMD transmission associated with the movement of beef produced under such conditions was low but not negligible. However, it is also true that the FMD risk for animals arriving at slaughter is likely (1) less than 100%, even in endemic settings, (2) varies across regions and production systems, and (3) may be mitigated by certain measures. A first objective of exporting markets may be to sufficiently decrease the risk at slaughter, so that deboning and processing would result in negligible levels of risk. For that reason, in order to achieve and demonstrate a level of risk acceptable to many trade partners, it is necessary to extend that post-harvest risk assessment [21] to consider the risk of infection among animals arriving at slaughter from local systems. The relative risk of FMD infection among livestock exported from Somalia has been modeled for several scenarios to compare the impact of risk reduction strategies [24]. They found that cattle held in 21-day quarantine at the point of export and inspected daily had 4% of the risk of being exported while infected with FMD compared to cattle exported with no control measures implemented.

Although previous studies have assessed the epidemiology [25,26,27], risk factors [28,29,30], and challenges of FMD control [9,11,12] in Kenya and Uganda, the risks for FMD in cattle and deboned beef originating from both countries have not been quantified. Infection risk among animals at slaughter is dependent on the events that occur between the farm and abattoir in addition to the herd-level disease risk [21], especially considering the important risk presented by animals in the early incubation phase of disease [31]. Kenya and Uganda each have several beef cattle production systems [32,33,34] and complex ruminant value chains [35]. In order to complete a risk assessment that can usefully guide each country toward steps to reduce risk, it is important to include information about the distinct risk factors associated with the production, sale, and transport of beef cattle from each management system.

Risk is a concept that incorporates both the probability of occurrence of an event and the magnitude of the consequence if the event does occur. In this publication, the terms risk and probability are used interchangeably unless otherwise specified, always referring to the probability that a given event takes place without evaluation of consequence. A complete import risk analysis, from the importer perspective, would consider the magnitude of the consequences if the event occurs in order to guide decisions. Exporters consider what it would take (what measures and at what cost) to appease potential importers, and, given those concessions or investments, if the product would be competitive in that market, profitable for local producers, and a worthwhile pursuit for public and private resources.

The objective of this study was to estimate the probability of FMD infection among cattle at the time of slaughter originating from eight total production systems in Kenya and Uganda using a stochastic risk assessment model. Results showed a wide gap in Kenya between systems at high (pastoral, semi-intensive) and low (feedlot, ranching) risk. By contrast, in Uganda, all systems had similar values for total probability despite differences in individual inputs and nodes. Model results indicate that this probability could be reduced by varying degrees in all systems by eliminating or even reducing commingling with other cattle between sale and slaughter. The next step in contextualizing these results is to consider specific interventions that may reduce that probability to a level acceptable to trading partners and the cost, logistics, and tradeoffs of each. The potential costs and benefits of pursuing those interventions to participate in international trade can then be weighed in light of the opportunities and capacity of each country.

## 2. Materials and Methods

### 2.1. Model Overview

#### 2.1.1. Setting and Production Systems

Beef cattle production systems in Kenya and Uganda have been classified by the Food and Agriculture Organization of the United Nations (FAO) through a process that engaged key national stakeholders and synthesized sources of cattle distribution and production data [33,34]. These classifications were reviewed by Veterinary Services (VS) members for their country and evaluated as appropriate to use for classifying risk assessment inputs and assessing results [36]. The four production systems in Kenya are: feedlot (1% of beef cattle), pastoral (34%), ranching (11%), and semi-intensive/agropastoral (54%). The four production systems in Uganda are: agropastoral (49%), pastoral (41%), ranching (8%), and semi-intensive (2%). 

#### 2.1.2. Risk Question and Model Formulation

A quantitative and stochastic risk model was developed to estimate the baseline risk of the slaughter of FMD-infected cattle from distinct production systems in Uganda and Kenya. The question to be answered for each of four cattle production systems in two countries was: What is the probability that cattle sold for meat are slaughtered while infected with FMD virus (FMDV)? Specifically, the outputs of interest for the model were:

- the probability for any cattle sold for meat to be slaughtered while infected
- the annual probability that at least one infected bovine is slaughtered

**Figure 1:**
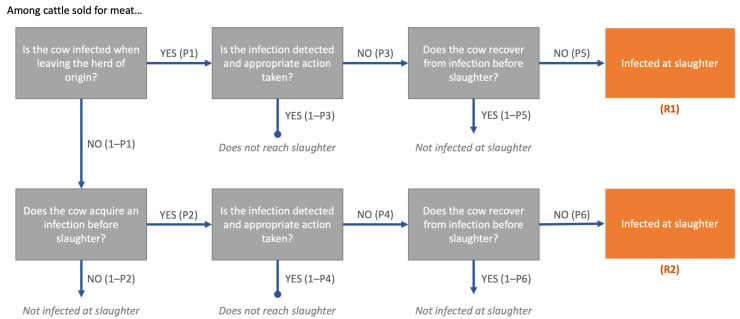
Risk pathways for the probability of FMD infection at slaughter among cattle sold for meat in Kenya and Uganda. *R*1 represents cattle infected at the time of leaving the source herd. *R*2 represents cattle that acquire new infections between the herd and the time of slaughter. The total probability, *Ptot*, is the sum of *R*1 + *R*2. Each event is conditional on the preceding events.

The major events and pathways resulting in the possible slaughter of an FMD-infected animal are depicted in Figure 1. The inputs and probabilities are described in the following section and summarized in Table A1. The input variables and relationships described were used to construct a stochastic risk assessment model. The model structure was the same for each production system and country; distinctions were represented through differences in input variable distributions.

A stochastic model uses input distributions rather than point estimates for some or all input variables and calculates a probability distribution of the possible model outcomes based on the combined impact of the variation of each input [37]. The following paragraphs describe how those distributions, representing the total uncertainty and variability for each input variable, were derived. Each input (including the distribution used to represent that variability) and calculation is described in Table A1.

Two pathways were identified through which the event of slaughtering an FMD-infected animal may occur—in which the animal is infected in the herd of origin (already infected at the time of sale) or through contact with infected animals between the farm and slaughter. The probability that a single animal sold is slaughtered while infected with FMD virus through each of the respective pathways is given by:

*R*1: Infected on farm before sale, not detected and does not recover: *P1 ∗ P3 ∗ P5*

*R*2: Infected after sale, not detected and does not recover: *(*1 − *P1) ∗ P2 ∗ P4 ∗ P6*

Where each *P* is the conditional probability associated with that step, given each of the previous steps in the pathway.

The probability that cattle sold for meat are slaughtered while infected with FMDV (*Ptot*) via either pathway is the sum of *R*1 and *R*2:

*Ptot* = *R*1 + *R*2

The probability that at least 1 bovine sold for meat reaches slaughter while infected can be calculated as a binomial process:

$$Pany=1-{\left(1-Ptot\right)}^{N}$$

where *Pany* represents the probability of at least 1 event occurring. 

The pathways outlined above follow standard logical and probability relationships used in risk modeling [37,38].

### 2.2. Evidence Gathering and Parameter Estimation

The populations and key processes, variables, and relationships were identified in partnership with mid-career veterinarians in Kenya and Uganda. The elicitation process and outputs have been described elsewhere [36,39]. A probability distribution for each input variable for each population was described using information available through scientific literature, country reports, and the opinion of professional veterinarians in each country. 

#### 2.2.1. Are Cattle Infected When Leaving the Herd of Origin? (*P1*)

The prevalence of FMD among cattle sold from each management system was calculated as the annual probability of infection per head times the probability that an infected animal would be sold divided by the probability that any animal (infected or uninfected) would be sold. This formulation was used because VS members indicated that the probability of infection among animals sold should not be assumed to be the same as the probability of an animal chosen at random from the source population, and so prevalence in the population is not an appropriate proxy for prevalence among cattle sold. 

Because all values used the same denominator (total population times 365 days/year), the calculation was simplified to:

*P1* = *C* ∗ *Si*/*S*,

where *C* is the number of infections in the population per year, *Si* is the probability that an infected animal is sold while infected, and *S* is the number of sales from the population per year. 

The annual number of FMD infections in each management system (*C*) was estimated from cross-sectional seroprevalence data collected in each country as well as the mean age and total population of cattle in each system. 

Distributions of annual incidence in Uganda were based on data reported elsewhere [27,29]. A total of 14,439 cattle from 211 herds were tested for antibodies to non-structural FMDV proteins using a PrioCHECK ELISA test kit (Thermo Fisher Scientific, Waltham, MA, USA). More details about the sample collection, processing, and testing are available elsewhere [27,29]. The data used for this analysis was limited to animals chosen as part of random sampling (not purposively targeted) and with age at time of sampling between six months and three years of age (n = 3468 individuals from 111 herds). The mean and standard error for the proportion of positive animals, accounting for clustering within herd and regional-level sampling weights [40] (using Stata (version 16, College Station, TX, USA)), were used to construct a beta distribution of the prevalence of antibodies against FMD virus (*Pr*) within each of the four production systems in Uganda. Because a positive ELISA result represents at least one seroconversion event within the animal’s lifetime, the prevalence was divided by the mean age of the respective population (*A*) to reach an estimate of the incidence of new infections per year in each production system. The distribution of mean age for each management system was built by bootstrapping from the ages of cattle sampled within each system, sampling with replacement at the herd level and calculating the mean age of each bootstrapped sample. 

Two alternative approaches to estimating the incidence of FMD in Uganda were evaluated for impact on the overall risk. In the default scenario, described above, all cattle with positive ELISA results for NSP antibodies were classified as having experienced an FMD infection. In the first alternate approach, cattle that were positive for antibodies and had a record of vaccination within six months of the date of testing were classified as FMD negative (evaluating the possibility that such animals had a vaccine-induced antibody response). All subsequent steps for estimating *P1* using these data were the same as described above. In the second alternate approach, virus isolation (VI) results from probang (oropharyngeal) samples taken on a subset of the cattle surveyed were used instead of ELISA testing. This dataset, limited to cattle of any age from randomly chosen herds, contained 488 cattle from 29 herds from only the Eastern and Northern regions of Uganda (region classification and more information on sample collection and processing available elsewhere [27]). The mean and standard error for the proportion of VI-positive animals, accounting for clustering within herd and regional-level sampling weights, were used to construct a beta distribution of the prevalence of virus in probang samples from each production system (semi-intensive and ranching systems were combined into one due to limited data). The annual incidence was calculated as the prevalence divided by the average duration of infection in days times 365 days per year. The average duration of infection was specified as a function of the probability that an infection was acute or persistent and the associated duration of an acute infection (*D*) or persistent infection (Pert distribution with minimum of six months, maximum of 24 months, and most likely value of 13 months [41,42,43]).

Distributions of annual incidence in Kenya were based on data reported elsewhere [26,44]. Serum samples from 2908 cattle in 39 counties in Kenya were ELISA-tested for antibodies to non-structural FMD virus proteins. More information about sample collection, processing, and testing are available elsewhere [26,44]. The management system was not recorded, so prevalence for pastoral, semi-intensive, and ranching production systems was estimated by restricting analysis to counties with at least 80% of cattle in pastoral systems (n = 10 counties), at least 80% of cattle in semi-intensive systems (n = 14 counties), and at least 50% of cattle in commercial ranching systems (n = 2 counties). Given the low sample size, an alternative parameterization was evaluated with a range between 0 and 1 and most likely value 0.47 (the mean of the prevalence in those two counties); there was no notable impact on the model output, so the default parameterization was retained. Because feedlot operations make up a small portion of total beef cattle and operations, the prevalence was given a range between 0 and 1 and results from a survey of 31 feedlots in Ethiopia [45] used to define the most likely value. The mean and standard error for the proportion of positive animals, accounting for sampling weights and stratification by county, were used to construct a beta distribution of the prevalence of antibodies against FMD virus (*Pr*) within each of the four production systems in Kenya using Stata (version 16, College Station, TX, USA). The prevalence was divided by the mean age of the respective population (*A*) to estimate the incidence of new infections per year. The distributions of mean age were based on the reported age of animals sampled [44] in counties with predominantly pastoral or semi-intensive animals and on reports relevant to ranching, pastoral, and feedlot systems [45,46].

The cattle population for each production system was calculated as the percent of cattle in each system (*Mg*) reported by the FAO classifications described above [33,34] times the national beef cattle population (*Np*). The national population in Kenya was estimated from descriptions ranging from 14.1 million to 16 million cattle raised for meat [17,33,47]. The national population in Uganda was estimated from descriptions that ranged from 12.1 million to 15.9 million head [47,48,49].

The probability of sale among infected animals (*Si*) was estimated by the VS participants as described elsewhere [36]. 

The number of cattle sold per year from each system (*S*) was calculated as the offtake rate times the cattle population. Estimates for annual offtake rate (*O*) within each production system were based on ranges reported by studies in Uganda, Kenya, and Ethiopia [46,50,51,52,53]. Feedlots were estimated to have one to four cycles of fattening per year with complete turnover of their population for each cycle, i.e., an offtake rate ranging from 100 to 400%. In Kenya, these estimates in each population amount to a mean national offtake rate of 17.2% per year, in alignment with the range of 15–20% calculated using FAOSTAT estimates. In Uganda, they add up to a mean national rate of 11.5%, compared to an estimated 12% reported in 1998 [54].

#### 2.2.2. Do Cattle Acquire a New Infection before Slaughter? (*P2*)

The probability of acquiring a new infection en route to slaughter was calculated based on the probability of mixing with cattle from other herds (1 − *Pn*) and subsequent effective contact with an infectious animal (*Ic*):

$$P2=\left(1-Pn\right)*Ic.$$

The probability of commingling was estimated through discussion with VS participants, in which they estimated the proportion of animals from each management system which do not commingle with cattle from any other herds before slaughter (*Pn*). 

The probability of effective contact (*Ic*) was formulated as a binomial process. Commingling was assumed to result in effective transmission if the animal mixed with was infectious with FMD virus. Therefore, the probability of at least one infectious contact among cattle that mix with other animals was defined as:

$$Ic=1-{\left(1-Pa*Pi\right)}^{Nm},$$

where *Pa* was the overall prevalence of FMD among animals sold (all management systems combined), *Pi* the probability that an infected animal is infectious, and *Nm* the number of cattle mixed with when commingling occurs. 

The probability that an infected animal is infectious (*Pi*) was based on the ratio of the latent (preinfectious) period (*L*) to the total duration of an acute infection (*D*). Distributions for the phase durations for the latent, incubation, and infectious periods of an acute FMD infection were each constructed by sampling from ten equally-weighted distributions: two from meta-analyses of experimental studies [55,56] and eight from a single study with distributions constructed from the input of 11–15 experts for scenarios combining high or low virulence, high or low virus dose, and airborne or direct contact transmission [57]. 

We assumed that all animals who are not infected upon leaving the herd are susceptible to new infections. 

#### 2.2.3. Is the Infection Detected and Appropriate Action Taken, among Cattle Infected on the Herd of Origin? (*P3*)

*P3* is the probability that cattle infected with FMD at the time they leave their herd of origin are not effectively detected and acted upon. Effective detection and action require that cattle are inspected (*In*), are displaying clinical signs at the time of inspection (*Cl*), and that the inspection identifies and reports the clinical inspection (*De*). Therefore, *P3* was defined as:

P3=1−In∗Cl∗De.

The probability that cattle are inspected at least once (*In*) was defined as one minus the probability that cattle completely bypass inspection as estimated by the VS participants of each country. 

The probability of displaying clinical signs at the time of inspection (*Cl*) was equal to the probability of being in a clinical phase of infection on a random day between sale and slaughter: defined as the ratio of the days of clinical infection remaining after the animal is sold from the herd to the duration of the whole process from herd to slaughter (*Dp*). Any values greater than one or less than zero were set to one and zero, respectively. Conceptually, there were three categories of values: cattle in which clinical signs start before the time of sale (*Cl* is equal to one); cattle in which clinical signs do not begin until after the herd-slaughter process is over (*Cl* is equal to zero); and cattle in which clinical signs develop sometime during the process (*Cl* is between zero and one). 

The probability that an inspected, clinically infected animal is effectively detected and reported (*De*) was specified as a binomial process based on the effectiveness of each inspection and the number of inspections received. The effectiveness of inspection was defined by VS responses and discussion as described [36]. Briefly, it was based on the sensitivity of inspection, the reporting rate of positive animals, and the ratio of two different levels of inspection quality (*W*1, *W*2). The number of inspections (*Ni*) was also described by VS participants. 

#### 2.2.4. Is the Infection Detected and Appropriate Action Taken, among Cattle That Acquire New Infections en Route? (*P4*)

*P4* is similar to *P3*, with a different value for the probability that an infected animal is displaying clinical signs at the time of inspection (*Cn*). *Cn* was defined as the ratio of days during which newly infected cattle are in a clinical phase of infection compared to the duration of the whole process from herd to slaughter (*Dp*). It was assumed that a new infection could be acquired with equal probability on any day during the process. *Cn* was adjusted to be bounded at zero and one as described for *Cl*.

P4=1−In∗Cn∗De.

#### 2.2.5. Do Cattle Infected on the Herd of Origin Recover from Infection before Slaughter? (*P5*)

Cattle infected on-farm were assumed to recover before slaughter if they had acute infections and the duration of infection remaining when leaving the herd was less than the duration of the herd-to-slaughter process:

P5=1−Pa∗Re,

where *Pa* is the probability that an infection is acute, and *Re* the probability of recovery from an acute infection.

The probability that an infection persisted beyond the acute stage was described with parameters from the literature. A review of the carrier state for FMD [58] described from 20% to over 50% of cattle likely to be carriers. In a short communication of animals slaughtered in Uganda [59], nine out of 12 animals slaughtered had viral RNA in the oropharyngeal tissue at slaughter three months after the lifting of quarantine measures. Therefore, the probability of acute infection was described as the complement of a PERT distribution with a minimum of 0.2, maximum of 0.75, and most likely value of 0.5. 

The recovery from an acute infection (*Re*) was specified as a Poisson process. The rate (*Rr*) was defined as the reciprocal of (one over) the duration of infection (*D*) and the exposure time (*Ro*) defined as the sum of the process duration (*Dp*) and the days of infection prior to the day of sale (*Ts*). Therefore, the probability of not recovering before slaughter among cattle with acute infections was defined as the probability that the event does not occur during that period of time, *exp(−Rr∗Ro).*

#### 2.2.6. Do Cattle Infected en Route Recover from Infection before Slaughter? (*P6*)

*P6* is similar to *P5*, with the exception that the exposure time for recovery (*On*) was defined as the difference between the duration of the herd-to-slaughter process (*Dp*) and the day of that process on which infection occurred (*Tn*).

P6=1−Pa∗Rn.

#### 2.2.7. Number of Cattle Exported Annually (N)

The number of cattle that would be exported annually was hypothetically assigned to be 20% of the total current production (*S*) from a given management system. 

Quantities *P1–P6* and *N* were combined to simulate distributions for *R*1, *R*2, *Ptot*, and *Pany* for each of the four management systems for Kenya and Uganda. 

### 2.3. Sensitivity Analysis

Sensitivity analysis was performed to identify the most influential nodes and input parameters and evaluate the impact of their uncertainty on the overall risk estimate, *Ptot*, within each production system. Each node value was divided into percentiles (1, 5, 25, 50, 75, 95, 99) and the conditional mean value of *Ptot* was calculated when the node was held fixed within each percentile interval while all others varied randomly (similar to the Change in Output Mean function of @Risk (Palisade Corporation, Ithaca, NY, USA)). For production systems with a median total risk less than 0.5, the input variables were also plotted and examined similarly.

### 2.4. Model Environment

All Monte Carlo simulations were performed using RStudio [60] and R software version 4.0.2 [61] to estimate the outcome distributions by computing 30,000 iterations of each model. Stata (version 16, College Station, TX, USA) was used to calculate the mean and standard error of the prevalence estimate in each management system while accounting for clustering, stratification, and sampling weights [40]. 

## 3. Results

### 3.1. Total Probability

**Figure 2:**
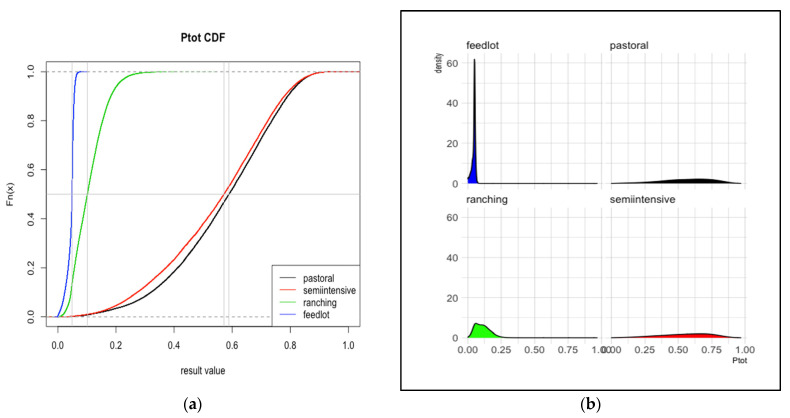
Total risk, Kenya. The cumulative distribution functions (**a**) and probability density functions (**b**) for the probability of cattle sold for meat arriving at slaughter while infected with FMD from each of four production systems in Kenya. The vertical gray line represents the median value. Distributions are based on 30,000 iterations of the stochastic model. *Ptot* is the sum of *R*1 and *R*2 depicted in Figure 1.

**Figure 3:**
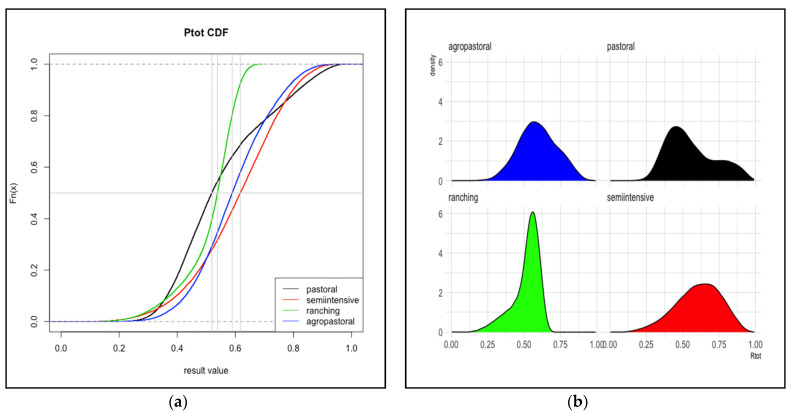
Total risk, Uganda. The cumulative distribution functions (**a**) and probability density functions (**b**) for the probability of cattle sold for meat arriving at slaughter while infected with FMD from each of four production systems in Uganda. The vertical gray line represents the median value. Distributions are based on 30,000 iterations of the stochastic model. *Ptot* is the sum of *R*1 and *R*2 depicted in Figure 1.

The probabilities of FMD infection at slaughter, estimated for cattle from eight total production systems in Kenya and in Uganda are reported in Table A2. Plots of the cumulative distribution function and probability density function for overall probability (*Ptot*) are shown in Figure 2 and Figure 3.

In Uganda, the overall probability that cattle arrive at slaughter while infected was similar across all four production systems (despite substantial variation between systems in the values for the pathways of being sold while infected, *R*1, and acquiring a new infection en route to sale, *R*2). Ranching had the lowest mean *Ptot* at 0.52 (95% interval: 0.27–0.64), followed by pastoral with mean 0.55 (0.31–0.91). The mean probability (95% interval) for agropastoral and semi-intensive systems was 0.59 (0.35–0.85) and 0.61 (0.28–0.87), respectively.

In Kenya, there was a sharp demarcation between two groups of systems (in contrast to Uganda). Those with lower *R*1 also had lower *R*2 values, so the sum of those, *Ptot*, compounded the gap. Feedlots (mean 0.04, 95% interval 0.01–0.06) and ranching systems (mean 0.11, 95% interval 0.03–0.23) had a relatively low overall risk. Pastoral and semi-intensive systems had high *Ptot* values, with mean values of 0.57 (0.17–0.85) and 0.55 (0.16–0.85), respectively.

The probability of at least one infected animal slaughtered per year (*Pany*) had a 95% interval spanning from 1 to 1 for each of the systems evaluated, given the estimated exports volume of 20% of total sales from a given system. In other words, there is 95% confidence of the occurrence of at least one infected animal at slaughter in a given year under current conditions from each of the production systems modeled.

### 3.2. Influential Variables and Nodes

The analysis highlighted two groups of production systems according to which pathway contributed most to the overall risk. Most management systems (Kenya: pastoral, ranching, semi-intensive; Uganda: agropastoral, ranching, semi-intensive) were *R*2-dominant: the expected value of the *R*2 pathway was higher than *R*1, and correspondingly the value for the node *P2* (probability of acquiring a new infection en route) was greater than *P1* (probability of being infected at the time of sale). In other words, the greatest contribution to the total risk of infection at the time of slaughter was through new infections acquired between sale and slaughter. The other two systems—ranching in Kenya, and pastoral in Uganda—had a higher value for *R*1 than for *R*2 (and for *P1* than for *P2*).

**Figure 4:**
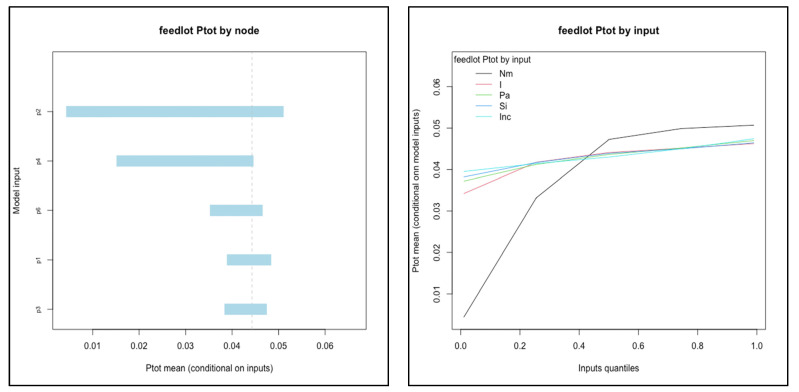

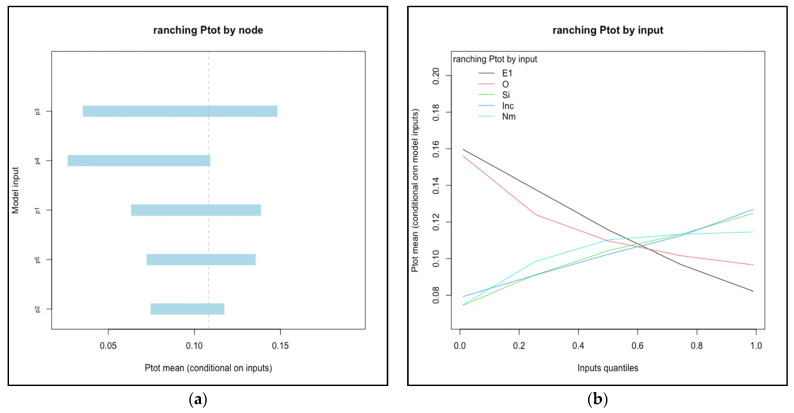
Influential nodes (**a**) and input variables (**b**) for feedlot and ranching systems in Kenya. Each node (*P1–P6*) or input value was divided into percentiles (1, 5, 25, 50, 75, 95, 99) and the conditional mean value of *Ptot* was calculated when the node was held fixed within each percentile interval while all others varied randomly. Only the top five most influential nodes or inputs were included for each plot. Note that the axes vary for each.

For systems below a threshold risk (median *Ptot* less than 0.50), the relationships of input values to conditional mean output were evaluated in order to identify candidate variables for interventions that may reduce total risk into a range likely to be acceptable to potential trade partners. The two systems that met the criteria were feedlot and ranching systems in Kenya (see Figure 4). In feedlot systems, *P2*(probability of acquiring a new infection en route) and *P4* (probability that a new infection is not detected) were the most influential nodes. The number of cattle mixed with (*Nm*) was the single most influential input variable: mean *Ptot* ranged from 0.004 to 0.05 as *Nm* increased from the 1st to 99th percentile values (0 to 112 animals mixed with). In ranching systems, *P3* and *P4* (probability of not detecting an infection that originated on-farm and en route, respectively) were the nodes associated with the largest range of conditional mean values for *Ptot* (from 0.03 to 0.15 as *P3* increased from 1st to 99th percentile values). Efficacy of inspection (*E*1) was the most influential input variable.

### 3.3. Alternative Approaches

**Figure A1:**
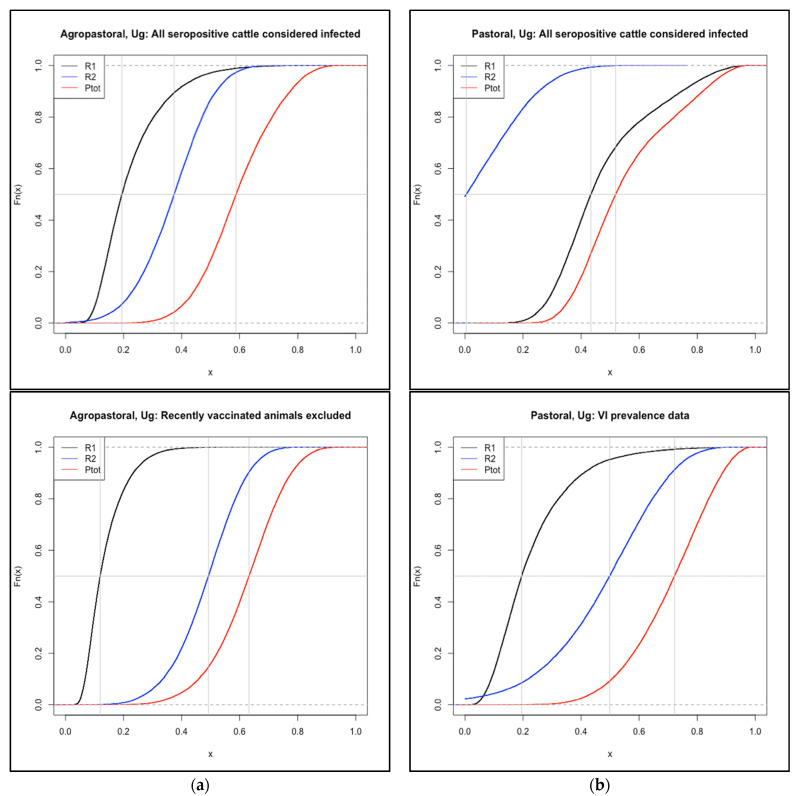
Sensitivity analysis: Reductions in *P1* result in “paradoxical” increases in total risk. Cumulative distribution functions for *R*1 (cattle infected in the source herd), *R*2 (cattle infected en route to slaughter), and *Ptot* (overall probability of infection at slaughter) for two production systems in Uganda under the default and alternative approaches to estimating FMD incidence. Top left: agropastoral, default. Bottom left: agropastoral, animals seropositive with record of vaccination within six months of sampling are called FMD-negative. Top right: pastoral, default. Bottom right: pastoral, viral isolation from probang samples used rather than serology. Vertical gray lines indicate the median value of each curve. (**a**): Under the scenario where antibodies of recently vaccinated animals are assumed to indicate vaccination rather than infection (bottom panel), the agropastoral system has the largest decrease in prevalence of all systems. This resulted in a reduction in the median value of *R*1 to 0.14 (from 0.22 in the default scenario (**top**, black curve)). An increase in *R*2 (due to more animals eligible for infection) “compensated” for the lower *R*1, and the median *Ptot* was slightly higher in the alternative scenario despite the lower prevalence. (**b**): Where viral isolation data were used, rather than serology to estimate the annual incidence of disease, the pastoral system had the largest decrease in prevalence of all systems, causing the median *P1* value (not shown) to drop to 0.40 (from 1.0 in the default scenario). The lower prevalence reduced the median value of *R*1 from 0.43 to 0.20. The resulting increase in *R*2 “compensated” for the lower *R*1, and the median *Rtot* was higher in the alternative scenario due to the impact of new infections acquired during the sale process, despite the lower estimated occurrence of disease in the source population.

Two alternative approaches for the estimation of FMD prevalence among Uganda cattle populations were evaluated (Figure A1). Under the first approach, where antibodies of recently vaccinated animals were assumed to indicate vaccination rather than infection, the agropastoral system had the largest decrease in prevalence of all systems and resulted in a reduction in the median value of *R*1 (probability of infection at slaughter due to cattle infected when leaving the source herd) to 0.14 (from 0.22). An increase in *R*2 (new infections acquired en route, due to more animals eligible for infection) “compensated” for the lower *R*1, and the mean *Ptot* was slightly higher (0.62 vs. 0.59 in the default) in the alternative scenario despite the lower prevalence. Where viral isolation data were used rather than serology to estimate the annual incidence of disease, the pastoral system had the largest decrease in prevalence of all systems, causing the median *P1* value to drop to 0.40 (from 1.0 in the default scenario). The lower prevalence reduced the median value of *R*1 from 0.43 to 0.20. The resulting increase in *R*2 “compensated” for the lower *R*1, and the mean *Rtot* was actually higher in the alternative scenario (0.7 vs. 0.55 in the default) due to the impact of new infections acquired during the sale process, despite the lower estimated occurrence of disease in the source population.

In both cases, any large change to the estimation of disease occurrence and thereby *P1* led to a decrease in the value of *R*1. However, since *R*2 includes the value *(*1 − *P1)*, there was a compensatory effect (smaller *P1* values resulted in larger *R*2 values) and even a paradoxical increase in the overall risk, *Ptot*.

## 4. Discussion

In this study, we modeled the risk of FMD infection among cattle at the time of slaughter for cattle originating from four different management systems under current conditions in Kenya and in Uganda. These values and relationships provide an essential input for further evaluation of marketing and risk management considerations, although a full analysis requires more than the probability of occurrence.

As well as providing quantitative knowledge about the FMD risk, this work provides a framework that can be adapted to quantify the cost-effectiveness of specific strategies, in specific farms or groups of farms if needed. For example, the impact of an intervention targeting specific clusters of farms could easily be measured by modifying the probabilities in the nodes. This work is a required step in transitioning from qualitative descriptions of the setting [36] to specific assessments intended to evaluate the cost-effectiveness of interventions. The novelty of the work here is that such transition has never been published for implementation in the East Africa setting.

The first step in contextualizing these results will be to consider interventions that may reduce that probability to a level acceptable to trading partners along with the cost, logistics, and tradeoffs involved in each. The risk estimates and sensitivity analyses produced here provide insight about influential factors that could be leveraged to effectively lower the probability of FMD among beef cattle at slaughter from select populations.

Our results highlight the heterogeneity between countries and even between production systems of risk for FMD among cattle at slaughter. In Kenya, a wide gap emerged in the total risk between systems at high (pastoral, semi-intensive) and low (feedlot, ranching) risk. By contrast, in Uganda, all systems had similar values for total risk despite differences in individual inputs and nodes. This distinction between countries was driven in part by the wide gap in *P2* values (new infections en route) among Kenyan systems, due to the probability of completely avoiding mixing with other animals as described by Veterinary Service professionals—0.95 for feedlot and ranching, 0.05 for pastoral and 0.10 for semi-intensive. In Uganda, all systems had a fairly low probability, or a wide range that included low probabilities, of avoiding commingling. Therefore, *P2* values were modestly high (mean value greater than 0.5) for all systems in Uganda. In both countries, *P3* through *P6* (the probability of non-detection and recovery for cattle infected on-farm or en route) were similar between systems (mean value within 0.10 range) and the distinction of total risk between systems was driven by the diversity of *P1* and *P2* values. Thus, the differences in commingling probability played a key role in separating the Kenyan systems (and, likewise, failing to separate those in Uganda). The variation between regions and systems is important because of the possibility to focus investments and interventions in targeted populations for which exporting beef is a feasible and favorable opportunity.

Feedlots in Kenya had the lowest risk among all production systems assessed in both countries, driven by low values for both infections among animals sold (*P1*) and new infections acquired en route (*P2*). Feedlots are finishing systems where cattle from ranches or pastoral systems spend between three months and a year for fattening and are sold through formal channels to prime or niche markets. There are very few feedlots in Kenya (1% of cattle farms [33]). Feedlots require high levels of input (capital and labor) and typically invest in relatively robust biosecurity and animal health practices. The feedlot model requires a market that will pay a high price in order to be profitable, and has been historically limited by the low availability and high cost of feed inputs in Kenya [62,63]. If such a market could be secured/established and the feeds issue solved, feedlots could be an option to increase the quality and consistency of beef produced from pastoralist and ranching value chains. Feedlots have been suggested as one strategy to mitigate the volatility of rainfall and temperature associated with climate change in the semi-arid areas of northern Kenya [64].There are few data on the prevalence of FMD at feedlots in Kenya, so the range of possible values specified for the model (ranging from 0 to 1) reflected a great deal of uncertainty about disease occurrence. Regardless, the high rate of sales (assumed 100–400% turnover rate annually) diluted the impact of positive cases on the probability that any individual animal would be infected at the time of sale. The high offtake rate combined with the low probability of commingling as described by VS created the low overall probability of infection at slaughter as calculated by the model. While the low result may partially be a product of the dilution impact of such a high rate of sales and turnover, it is also true that such systems enable concentrated use of resources for disease prevention, surveillance, and documentation. The companion question is whether the beef produced from such high-input systems could be competitive and profitable on the international market.

Further reduction of FMD prevalence at slaughter for cattle originating from feedlots could be most effectively achieved by targeting nodes *P2* (new infections) and *P4* (detection of new infections), and specifically the number of animals mixed with when commingling occurs (*Nm*), according to our sensitivity analysis. Theoretical interventions that target these nodes are similar to measures already being discussed and implemented for some systems in Kenya [17,35]: direct shipment of cattle from their herd of origin to the point of slaughter and holding cattle in quarantine or holding areas where they are not exposed to cattle from other sources. If these measures were consistently implemented so that the risk of acquiring new infections en route was reduced or even eliminated, then additional biosecurity and animal health investments for disease prevention and control at the source herd could directly translate to lower probability of infection among cattle at the time of slaughter. 

These principles apply generally to many of the other, “higher-risk” management systems in our analysis, though the probability level achieved would not be as low as that predicted for feedlots. Elimination or reduction of the *R*2 pathway (infected slaughter cattle due to new infections en route) would be very effective to lower the prevalence of FMD-infected animals at slaughter, especially among cattle coming from a source population with a relatively low rate of infection. Most remarkably, if the *R*2 pathway were eliminated for ranching and semi-intensive systems in Uganda, the remaining *R*1 pathway (through cattle infected at the time of sale) would have a median total risk of 0.02. The most impactful strategy for any *R*2-dominant system would be to eliminate commingling completely; our model showed that even incremental decreases in exposure to other animals en route (fewer animals mixed with, or lower proportion of cattle who mix with other animals) can powerfully influence the prevalence of disease among animals at slaughter. Where that is not possible, an increase in the total length of the process from herd to slaughter could allow time for detection and/or recovery from infection (nodes *P3* through *P6*) but would also allow ongoing transmission of disease between animals if groups are not separated. Incubating, early stage infections among animals at the time of slaughter are especially concerning [21], as they are able to evade detection if not yet showing clinical signs, and viremia present at this stage is associated with viral particles in the skeletal muscle [31,65]. The international standards of the OIE for cattle originating from FMD-endemic regions recommends at least 30 days of holding animals in a quarantine station or FMD-free facility followed by direct transport to the abattoir for slaughter [20]. A risk analysis in Somalia [24] identified outbreaks within holding areas as an important source of infection among cattle who may have been disease-free when leaving their herd of origin. 

Cattle from Kenyan ranching systems were the second lowest in terms of infection probability at slaughter (median *Ptot* = 0.10). This was one of only two systems (along with Ugandan pastoral) in which probability associated with the *R*1 pathway (infected at the time of sale) was greater than *R*2 (acquired en route). Effective detection (*P3, P4*) were the nodes with greatest impact on mean risk (see Figure 4). Efficacy of inspection (*Eff*1) was the single most influential input variable for ranching systems in Kenya. Inspection quality is connected with other dynamic factors including volume of animals sold, availability of inspectors, and incentives for human actors to avoid corrupt behavior [36,66]. Our results indicate that ranching systems are capable of achieving a relatively low probability of infection at slaughter among cattle sold, and further study into the factors that could improve the consistency and quality of inspection within the existing value chains and infrastructure may elucidate ways to further reduce that level of risk. However, even at the highest values of *Eff*1 evaluated (inspection efficacy of 0.999), the mean risk (*Ptot*) was estimated to be 0.076. A similar risk reduction could be achieved if the *R*2 pathway was eliminated, as discussed for the systems above. (Median *R*1 value was 0.07; therefore, an *R*2 value of 0 would yield a median *Ptot* of 0.07). Thus, improvements in detection would need to be coupled with reductions in the opportunity for exposure and transmission to be most effective.

Another observation of note is that prevalence of disease in the source population (*Pr*) had relatively little influence in determining the mean value of overall probability of infection at slaughter (*Ptot*) for any production system. (The maximum impact was in Kenyan ranching systems, where the conditional mean *Ptot* associated with the lowest percentile of annual cases per animal (0.099) was a 27% reduction of the overall mean––see Figure 4). In fact, the analysis of alternative approaches demonstrated the futility of lowering prevalence in systems with opportunity for transmission of new infections before reaching slaughter (see Figure A1). This underscores the need to understand the specific goal (e.g., freedom from disease in a population versus risk reduction in a commodity) and the value of achieving that goal in a given system before investing scarce resources in the pursuit of development and health. 

Results and conclusions of this study should be interpreted in light of a number of limitations and assumptions. The management systems with the lowest risk were also the least common systems in each country (feedlot and ranching in Kenya, ranching and semi-intensive in Uganda). Consequently, there were fewer available data—published and VS experience—for these systems. Additional work to describe the production, health, and value chain dynamics of specific systems can confirm and clarify findings from this study about opportunities for impactful intervention and the level of achievable risk. A modeling study is only as reliable as its input data, and so these limitations as well as any sources of political influence on data sources should be considered when interpreting model results.

Assumptions in the risk model structure are also important to consider. It was assumed that all cattle who were not infected at the time of sale were eligible to be infected en route (i.e., no resistance). This is a conservative assumption as some cattle may have protection due to natural infection or vaccination. However, delivering a vaccine of adequate quality that is matched to circulating strains with appropriate frequency (recommended twice annually) is challenging. There are at least four serotypes of circulating FMD identified in East Africa with no cross-protection [27], such that infection with one may not confer resistance to other FMD viruses. In 179 serotype O viral isolates recovered from 48 herds of cattle in Uganda, only 1.1% were within the same topotype as the serotype O vaccine strain used in Uganda (topotype EA-1) [27]. Studies of vaccine coverage and efficacy in target populations could be integrated into the analysis to evaluate the impact of vaccination or assumptions about natural resistance. However, it is established that currently available FMD vaccines do not prevent subclinical infections [58] and an analysis integrating vaccination should also account for impacts on the probability of displaying clinical signs and of transmission to other cattle. 

The input values for cases (*C*), sales (*S*), and probability of sale given infection (*Si*) resulted in impossible values of *P1* (i.e., >1) for some iterations in some systems (most notably pastoral systems in Uganda). These values were forced to 1 for the model. The inputs were obtained from distinct sources and the tension highlights that the absolute value of *Si* may be less important than the relationship between the probability of sale for an animal that is infected and the probability of sale for any other animal (e.g., two times more likely to be sold? Or maybe only one third as likely to be sold?). The explicit relationship between the probability of sale in the two populations, and/or obtaining estimates for the probability of sale for each from a common source, would be useful in future analyses. Furthermore, this analysis did not incorporate temporal or spatial trends in events such as infection, sale, or movement of cattle that could potentially influence the range and shape of output distributions. Finally, the analysis did not account for clustering in the exposure of cattle to infection (either a group of animals sold together who had all been exposed on-farm, or clustering of exposures that occur at sale or during transportation). 

In summary, cattle from the Kenyan systems of feedlots and ranches had the lowest risk of being infected with FMD at the time of slaughter out of all eight systems evaluated. Model results indicate that this probability could be reduced by eliminating the commingling with other cattle between sale and slaughter; improved detection of infected animals was also indicated for ranching systems. For ranching and semi-intensive systems in Uganda, the risk of acquiring new infections en route raised the probability of infection at slaughter from a similarly low level (median risk less than 0.05) to approximately ten times higher; a reduction or elimination of that pathway could have substantial impact. Both Kenya and Uganda have published intentions to construct holding grounds and quarantine stations that would facilitate the export of livestock, meat, and leather from a consistent and high-quality cattle supply [17,67]. Our analysis indicates that such an approach—utilizing cattle from existing ranches in a feedlot/finishing type system with high biosecurity measures and following strict isolation from other cattle populations until slaughter—would capture the most effective risk reduction strategies from the viewpoint of reducing the probability of FMD infection among cattle at slaughter. 

However, such ambitious plans can be challenging to implement in reality. The insights from this analysis can contribute to formulating steps that may help move specific populations toward production of beef with a lower probability of FMD transmission through trade. These results need to be contextualized further, including understanding how low of a probability would be necessary to achieve certain trade benefits, what else would need to be done for market success once that level is achieved (is FMD the true bottleneck?), and the cost of implementation for risk management interventions and other measures needed to achieve those risk reduction targets and benefits. An important alternative to evaluate would be participation in markets where FMD is not an automatic barrier to trade, including livestock deficit countries within the COMESA (Common Market for Eastern and Southern Africa) preferential trade area of which Kenya and Uganda already participate [17]. In that scenario, FMD would be managed as an obstacle to health and productivity but without the extreme measures required for entry to more premium markets. In several assessments of other African countries, the expected benefits of removing the FMD barrier to entry to premium international beef markets, through either disease-free compartments or commodity-based trade, have not automatically justified the investment required [68,69,70]. Pressure to achieve disease freedom and control in Africa for trade purposes has historically been driven by European interests [12,71] and it is important that analyses to guide the investment of scarce resources for health and development are grounded in the values and capacity of each country even if the resulting steps forward are more modest.

## Data Availability

The data utilized for the study can be found in references cited in Table A1. The estimates generated through expert elicitation with Veterinary Service participants are described at: https://hdl.handle.net/11299/223215 (accessed on 24 November 2021).

## Appendix A

**Table A1:** Input variables, values, and references for the stochastic risk assessment model to evaluate the risk of FMD infection among cattle at the time of slaughter for animals sourced from four different production systems in each of two countries (Kenya and Uganda). Many but not all input variables had distinct values for each management system. F = feedlot, P = pastoral, R = ranching, S = semi-intensive, AP = agropastoral.

Input	Variable	Distribution or Estimate	Reference
Probability that a cow is infected when leaving the herd of origin	P1	$\frac{C*Si}{S}$ Adjusted for all values to be <1	NA
Number of FMD cases per year in source population	C	PrA∗Mg∗Np	NA
Probability that FMD-infected cattle are sold while infected	Si	Kenya: ~Pert (0.1, 0.2, 0.3) Uganda: ~Pert (0.2, 0.3, 0.4)	VS Estimates ^†^
Number of cattle sold for meat annually from the source population	S	O∗Np∗Mg	NA
Prevalence of antibodies against FMD non-structural proteins	Pr	Kenya, *~Beta (mean, sd):* F: Mode = 0.145, 95th pct = 0.9 P: (0.55, 0.01) R: (0.47, 0.06) S: (0.63, 0.02)	[26,44,45]
		Uganda, *~Beta (mean, sd):* AP: (0.29, 0.05) P: (0.72, 0.09) R: (0.09, 0.02) S: (0.09, 0.02)	[27,29]
Mean age of cattle surveyed for prevalence data	A	Kenya, *~Pert (min, mode, max):* F: (2, 4, 5) P: (2, 3, 5) R: (2, 2.5, 5) S: (2, 2.5, 5)	[44,45,46]
		Uganda, *Empirical distributions of mean age (mean, sd):* AP: (2.0, 0.04) P: (2.2, 0.06) R: (2.0, 0.06) S: (2.0, 0.06)	[29]
Proportion of total cattle population in each management system	Mg	Kenya, F: 0.01 P: 0.34 R: 0.11 S: 0.54	[33]
		Uganda, AP: 0.49 P: 0.41 R: 0.08 S: 0.02	[34]
National population of beef cattle	Np	Kenya: ~Pert (14100000, 14500000, 16000000) Uganda: ~Pert (12112000, 14189000, 15855000)	[17,33,47,48,49]
Percent of source population sold annually for meat	O	Kenya, *~Pert (min, mode, max):* F: (1, 3, 4) P: (0, 0.125, 0.25) R: (0.1, 0.24, 0.3) S: (0, 0.15, 0.25)	[46,50,51,52,53]
		Uganda, AP: (0.05, 0.1, 0.15) P: (0.05, 0.1, 0.15) R: (0.2, 0.25, 0.3) S: (0.2, 0.25, 0.3)	*Same as Kenya*
Probability that non-infected cattle acquire a new infection before slaughter	P2	(1−Pn)∗Ic	NA
Probability that cattle sold for meat do not mix animals from other herds before slaughter	Pn	Kenya, F: 0.95 P: 0.05 R: 0.95 S: 0.1	VS Estimates ^†^
		Uganda, *~Pert (min, mode, max):* AP: (0, 0.2, 0.5) P: (0, 0, 0) R: (0.3, 0.4, 0.5) S: 0, 0.25, 0.7)	VS Estimates ^†^
Probability that cattle who mix with others will experience at least one effective contact with an infected bovine	Ic	$1-{\left(1-Pa*Pi\right)}^{Nm}$	NA
Prevalence of FMD infection among all cattle sold	Pa	*Mixture distribution, unique for each country:* Mix(values = *P1*, probs = Mg)	NA
Probability that infected cattle are infectious on any day	Pi	1−LD	NA
Duration of latent phase (days pre-infectious)	L	*Equally weighted mixture of 10 distributions described in literature * Mean = 3.1, IQR = 1.4–4.1	[55,56,57]
Duration of total acute infection in days	D	L + I	NA
Duration of infectious phase	I	*Equally weighted mixture of 10 distributions described in literature * Mean = 8.6, IQR = 3.9–9.9	[55,56,57]
Number of animals from other herds commingled with, when mixing occurs	Nm	*~Nbinom (mean, IQR)* Kenya: (26.4, 9–36) Uganda: (18.4, 12–24)	VS Estimates ^†^
Probability that cattle infected at the time of sale are not detected and reported	P3	1−In∗Cl∗De	NA
Probability that cattle are inspected at least once between the source herd and slaughter	In	Kenya, *1—Pert(min, mode, max):* F: (0.01, 0.01, 0.05) P: (0.2, 0.4, 0.6) R: (0.01, 0.02, 0.05) S: (0.1, 0.2, 0.3)	VS Estimates ^†^
		Uganda, AP: (0.35, 0.4, 0.45) P: (0.4, 0.5, 0.6) R: (0.25, 0.3, 0.35) S: (0.1, 0.25, 0.4)	VS Estimates ^†^
Probability that cattle infected at the time of sale display clinical signs on a random day when inspection could occur	Cl	$\frac{Dp-Tc-Ts}{Dp}$ Adjusted for all values to be between 0, 1	NA
Duration in days of the process from leaving the source herd until slaughter	Dp	Kenya, *~Gamma (mean, IQR):* F: (1.1, 0.88–1.3) P: (9.0, 5.9–11.4) R: (1.2, 0.94–1.5) S: (6.5, 2.9–8.7)	VS Estimates ^†^
		Uganda, *~Lognormal (mean, IQR):* All: (2.5, 1.6–3.1)	VS Estimates ^†^
Day of infection on which cattle show clinical signs (Poisson process, time to first event)	Tc	$\mathrm{Exponential}(\frac{1}{Pc})$	NA
Day of infection on which cattle are sold	Ts	*Uniform* (0:D)	NA
Duration of incubation phase of infection (days pre-clinical)	Pc	*Equally weighted mixture of 10 distributions described in literature * Mean = 4.4, IQR = 2.5–5.7 Adjusted for all values to be ≤ D	[55,56,57]
Probability that inspected cattle showing clinical signs are detected and reported	De	$1-{\left(1-\left(W1*E1+W2*E2\right)\right)}^{Ni}$	NA
Probability that a “high quality” inspection detects and reports clinically-infected cattle	E1	*~Beta (mean, IQR)* Kenya: 0.70, 0.55–0.91 Uganda: 0.84, 0.78–0.92	VS Estimates ^†^
Probability that a “low quality” inspection detects and reports clinically-infected cattle	E2	*~Beta (mean, IQR)* Kenya: 0.52, 0.32–0.72 Uganda: 0.53, 0.44–0.63	VS Estimates ^†^
Proportion of high and low quality inspections experienced by cattle in each population	W1, W2	Kenya, F: 1, 0 P: 0.66, 0.34 R: 1, 0 S: 0.86, 0.14	VS Estimates ^†^
		Uganda, AP: 0.48, 0.52 P: 0.6, 0.4 R: 0.54, 0.46 S: 0.53, 0.47	VS Estimates ^†^
Number of times cattle are inspected between sale and slaughter, when inspected at least once	Ni	Kenya, *~Mixture(values, probs):* F: (1, 2) (0.25, 0.75) P: (1,2,3) (0.5, 0.33, 0.17) R: (1, 2) (0.25, 0.75) S: (1,2,3) (0.5, 0.33, 0.17)	VS Estimates ^†^
		Uganda, All: (1,2,3) (0.5, 0.33, 0.17)	VS Estimates ^†^
Probability that cattle infected between sale and slaughter are not detected and reported	P4	1−In∗Cn∗De	NA
Probability that newly-infected cattle display clinical signs on a random day when inspection could occur	Cn	$\frac{Dp-Tn-Tc}{Dp}$ Adjusted for all values to be between 0,1	NA
Day of sale-to-slaughter process on which cattle acquire new infection	Tn	$Uniform\left(0:Dp\right)$	NA
Probability that cattle infected at the time of sale do not recover before slaughter	P5	1−Pa∗Re	NA
Probability that infected cattle have an acute infection (not persistent)	Pa	1—*Pert* (0.2, 0.5, 0.75)	[58,59]
Probability that acutely-infected cattle recover before slaughter	Re	$1-Exp\left(-Rr*Ro\right)$	NA
Rate of recovery from acute infections (/day)	Rr	1D	NA
Duration during which acutely infected cattle have opportunity to recover before slaughter (days)	Ro	Dp+Ts	NA
Probability that cattle infected between sale and slaughter do not recover before slaughter	P6	1−Pa∗Rn	NA
Probability that newly-infected cattle recover before slaughter	Rn	$1-Exp\left(-Rr*On\right)$	NA
Duration during which acutely infected cattle with new infections have opportunity to recover before slaughter	On	Dp−Tn	NA
Number of cattle sold for export per year from each source population	N	0.2∗S	

**^†^** Full description and discussion of obtaining VS estimates available elsewhere [36].

**Table A2:** The median (25th, 75th percentile) values for each node (*P1–P6*), route (*R*1, *R*2) and total probability (*Ptot*) for each of four production systems in Uganda and in Kenya. The nodes correspond to events on the risk pathway as described in Figure 1. *R*1 *= P1∗P3∗P5. R*2 *= (*1 − *P1)∗P2∗P4∗P6. Ptot = R*1 *+ R*2.

**Median Values (25th, 75th Percentiles), Kenya**									
	***P1***	***P2***	***P3***	***P4***	***P5***	***P6***	***R*1**	***R*2**	***Ptot***
Feedlot	0.01 (0.00, 0.01)	0.05 (0.04, 0.05)	0.66 (0.28, 1.0)	1.0 (1, 1)	0.78 (0.70, 0.85)	0.98 (0.96, 0.99)	0.0 (0.00, 0.01)	0.05 (0.04, 0.05)	0.05 (0.04, 0.05)
Pastoral	0.29 (0.21, 0.41)	0.94 (0.80, 0.95)	0.74 (0.62, 0.88)	0.98 (0.88, 1.0)	0.63 (0.55, 0.71)	0.85 (0.74, 0.93)	0.13 (0.09, 0.20)	0.43 (0.27, 0.56)	0.59 (0.45, 0.71)
Ranching	0.15 (0.12, 0.19)	0.05 (0.04, 0.05)	0.65 (0.28, 1.0)	1.0 (1, 1)	0.77 (0.70, 0.84)	0.97 (0.95, 0.99)	0.07 (0.03, 0.11)	0.04 (0.03, 0.04)	0.10 (0.06, 0.14)
Semi-intensive	0.32 (0.24, 0.44)	0.89 (0.75, 0.90)	0.63 (0.43, 0.87)	1.0 (0.88, 1.0)	0.67 (0.59, 0.75)	0.90 (0.80, 0.96)	0.13 (0.08, 0.20)	0.41 (0.25, 0.53)	0.57 (0.41, 0.70)
**Median Values (25th, 75th Percentiles), Uganda**									
	***P1***	***P2***	***P3***	***P4***	***P5***	***P6***	***R*1**	***R*2**	***Ptot***
Agro- pastoral	0.44 (0.36, 0.53)	0.77 (0.70, 0.85)	0.59 (0.48, 0.88)	1.0 (0.97, 1.0)	0.74 (0.66, 0.81)	0.95 (0.91, 0.98)	0.19 (0.14, 0.28)	0.37 (0.29, 0.45)	0.59 (0.50, 0.68)
Pastoral	1.0 (0.83, 1.0)	1.0 (0.99, 1.0)	0.64 (0.56, 0.89)	1.0 (0.97, 1.0)			0.43 (0.35, 0.57)	0.0 (0.00, 0.14)	0.52 (0.43, 0.67)
Ranching	0.05 (0.04, 0.06)	0.59 (0.56, 0.62)	0.51 (0.38, 0.85)	1.0 (0.96, 1.0)			0.02 (0.01, 0.03)	0.51 (0.45, 0.55)	0.54 (0.48, 0.58)
Semi- intensive	0.04 (0.04, 0.06)	0.71 (0.61, 0.80)	0.48 (0.35, 0.84)	1.0 (0.96, 1.0)			0.02 (0.01, 0.03)	0.48 (0.59, 0.70)	0.62 (0.50, 0.72)
